# Detection of spontaneous preterm birth by maternal urinary volatile organic compound analysis: A prospective cohort study

**DOI:** 10.3389/fped.2022.1063248

**Published:** 2022-12-12

**Authors:** Emma Ronde, Nina M. Frerichs, Shauni Brantenaar, Sofia El Manouni El Hassani, Alfian N. Wicaksono, James A. Covington, Nanne K. H. De Boer, Tim G. De Meij, Thomas Hankemeier, Irwin K. M. Reiss, Sam Schoenmakers

**Affiliations:** ^1^ Division of Obstetrics and Prenatal Diagnosis, Erasmus University Medical Centre, Rotterdam, Netherlands; ^2^ Department of Pediatric Gastroenterology, Amsterdam University Medical Centre, Amsterdam, Netherlands; ^3^ School of Engineering, University of Warwick, Coventry, United Kingdom; ^4^ Division of Analytical Biosciences, Leiden Academic Centre for Drug Research, Leiden University, Leiden, Netherlands; ^5^ Department of Pediatrics, Division of Neonatology, Erasmus University Medical Centre, Rotterdam, Netherlands

**Keywords:** preterm (birth), volatile organic compound (VOC), biomarkers, metabolome, microbiome

## Abstract

Accurate prediction of preterm birth is currently challenging, resulting in unnecessary maternal hospital admittance and fetal overexposure to antenatal corticosteroids. Novel biomarkers like volatile organic compounds (VOCs) hold potential for predictive, bed-side clinical applicability. In a proof of principle study, we aimed to assess the predictive potential of urinary volatile organic compounds in the identification of pregnant women at risk for preterm birth. Urine samples of women with a high risk for preterm birth (≧24 + 0 until 36 + 6 weeks) were collected prospectively and analyzed for VOCs using gas chromatography coupled with an ion mobility spectrometer (GS-IMS). Urinary VOCs of women delivering preterm were compared with urine samples of women with suspicion of preterm birth collected at the same gestation period but delivering at term. Additionally, the results were also interpreted in combination with patient characteristics, such as physical examination at admission, microbial cultures, and placental pathology. In our cohort, we found that urinary VOCs of women admitted for imminent preterm birth were not significantly different in the overall group of women delivering preterm vs. term. However, urinary VOCs of women admitted for imminent preterm birth and delivering between 28 + 0 until 36 + 6 weeks compared to women with a high risk for preterm birth during the same gestation period and eventually delivering at term (>37 + 0 weeks) differed significantly (area under the curve: 0.70). In addition, based on the same urinary VOCs, we could identify women with a confirmed chorioamnionitis (area under the curve: 0.72) and urinary tract infection (area under the curve: 0.97). In conclusion, urinary VOCs hold potential for non-invasive, bedside prediction of preterm birth and on the spot identification of intra-uterine infection and urinary tract infections. We suggest these observations are further explored in larger populations.

## Introduction

Worldwide, preterm birth (PB) affects 15 million pregnancies annually and leads to significant neonatal morbidity and mortality (1). PB, defined as a delivery occurring before 37 + 0 weeks of gestation, can be either iatrogenic or spontaneous. Decades of investigation into the potentially treatable and preventable spontaneous PB (sPB) has shown a multifactorial origin with a still largely unraveled etiology and pathophysiology. sPB has been linked to maternal risk factors, most importantly obstetrical history, pregnancy characteristics, and uteroplacental risk factors including inflammation and infection (2). PB has been classified into the following categories by the World Health Organization: extremely preterm [less than 28 weeks of gestational age (GA)], very preterm (28–32 weeks of GA) and moderate to late preterm (32–37 weeks of GA) (1). Importantly, the earlier the PB, the higher the associated neonatal risks, with 95% of PB occurring after 28 weeks of GA (2, 3). The lack of identification of the exact mechanisms leading to sPB hampers the development of accurate prediction and prevention, resulting in increasing health related costs due to unnecessary hospital admittance and treatment (4–6). Maternal corticosteroids are used as antenatal treatment for women at risk for preterm delivery between 24 + 0 to 33 + 6 weeks of gestation and help reduce severity, frequency, or both, of respiratory distress syndrome, intracranial hemorrhage, necrotizing enterocolitis and death in neonates (7).

Recently, several studies have shown that unnecessary *in utero* exposure to maternal corticosteroids is associated with an increased risk for neurodevelopmental disorders later in life. For exposed children compared with unexposed children this risk was significantly different (12% vs. 6% with an absolute difference of 6% *P* < 0.001) and included decreased cognitive and behavior scores, an increased adjusted risk of sleeping disorders and a decreased adjusted risk of mild, moderate, or unspecified intellectual disabilities. In children eventually born late-preterm but antenatally exposed to corticosteroids, there was an association with a higher risk of neurocognitive disorders (adjusted hazard ratio 1.12, 95% CI: 1.05–1.20) and for children eventually born at term, there was a higher risk for mental or behavioral disorders (adjusted hazard ratio 1.47, 95% CI: 1.36–1.60) and suspected or proven neurocognitive disorders (adjusted hazard ratio 1.16, 95% CI: 1.10–1.21) (8, 9). This effect may be due to an altered programming of the hypothalamic-pituitary-adrenal axis in the developing brain, hypothetically because doses of corticosteroids used are actually higher than what is required for fetal lung maturation (9).

These results highlight the need for clinical applications to accurately/better determine which pregnant women and fetuses are actually at risk for PB (8–10).

Historically, sPB detection has focused on several approaches: (1) evaluation of maternal risk factors, (2) physical examination, including vaginal examination and ultrasound measurement of cervical length and (3) detection of biomarkers (11). Currently, besides the clinical presentation (contractions) and results of physical examination, determination of the fetal fibronectin originating from the vagina and cervix, seems the most promising biomarker (12). Fetal fibronectin is an extracellular matrix glycoprotein localized between the chorion and decidua. High levels in cervicovaginal secretions (greater than or equal to 50 ng/ml) have been associated with a greater risk of sPB with an overall sensitivity of 56% and specificity of 84%, however, measurements are subject to the gestational age at collection, population studied, single vs. multiple screening and the method of screening. It is therefore not yet recommended as standard work-up for prediction of sPB (13). Recently, nuclear magnetic resonance (NMR) spectroscopy and mass spectrometry (MS) have been used to study biomarkers associated with PB, such as prostaglandins (14, 15). Another promising biomarker technology comprises volatile organic compounds (VOCs) analysis, making use of the difference between physiological and pathological states based on gas and vapor-sensing (16).

VOCs are the end-products of metabolic processes, and an alteration of a metabolic state, as occurs during disease, will thus result in an alteration in the production of VOCs. In addition, metabolic processes also give insight into the genetic make-up of the individual's microbiome (17). Certain disease states, such as colorectal cancer and neonatal necrotizing enterocolitis are associated with different VOC profiles than detected in the physiological state (18–20). Also, UTI seem to be associated with specific urinary VOC profiles that can be used to discriminate between UTI and non-UTI (16, 21). Another recent study used VOC analyses in the exhaled breath of pregnant sheep to diagnose intra-uterine infections (22). Chorioamnionitis was further detected using VOC profiles in the amniotic fluid of time-mated ewes (23). Uncovering microbial pathways associated with specific VOCs in sPB in humans could provide more insight into the pathophysiology of PB and could hypothetically be used as predictive biomarkers (22). Since urinary collection and analyses are already part of the standard workup for pregnant women with a high risk for sPB, the use of urinary VOC profiles would provide for an easy, accessible and non-invasive technique.

Therefore, the primary objective of this proof of principle study, was to determine whether urinary VOCs can be used as predictive biomarkers to differentiate between pregnant women suspected for sPB who will actually deliver preterm and need admission and treatment from those who will eventually deliver at term (normal pregnancy). The secondary objective was to determine whether urinary VOCs can be used as predictive biomarkers to distinguish between pregnant women with an sPB associated infection, such as a UTI or chorioamnionitis, from those without.

## Material and methods

### Study design and participants

Inclusion criteria of this prospective, single-center cohort study were pregnant women [gestational age (GA) ≧ 23 + 5 weeks and <37 + 0 weeks] presenting with suspicion of imminent preterm birth [defined, according to the Dutch guideline for preterm birth, as a cervical length <25 mm as measured by vaginal ultrasound (24) and/or regular painful contractions and/or objective cervical changes such as effacement and/or dilation] who were admitted to the maternity ward of the department of Obstetrics at the Erasmus MC Sophia, Rotterdam, The Netherlands from July 2018–April 2019. Exclusion criteria were a pregnancy of a fetus with congenital disorders, history of cervical surgery, a twin pregnancy, delivery <24 + 0 weeks GA, lack of sufficient urine collection for VOC analysis and women unable or unwilling to give written informed consent for this study. At admission, the included women were physically examined for signs of preterm delivery, determination of infection parameters in blood [c-reactive protein (CRP), leukocytes], and temperature, while urine and vaginal swabs were collected and sent for culture. All women received tocolytics and antenatal corticosteroids aimed at improving fetal lung maturation according to local protocol (between 23 + 5 weeks until 33 + 6 weeks of GA). In case of preterm delivery, the placenta was sent for histopathological examination as standard of care. When women did not deliver, they were discharged and follow up was performed at the outpatient clinic of the department of Obstetrics of the Erasmus University Medical Center or the referring hospital. Baseline characteristics such as maternal age, ethnicity, BMI, intoxications, medical history, obstetrical history, use of medication (antibiotics, tocolytics and antenatal corticosteroids), details of delivery (mode of delivery, gestational age at delivery) and pregnancy complications (such as preterm rupture of the membranes and infection) were collected from the electronic patient file.

The study received exemption for approval from the local institutional Medical Ethics Committee according to the Dutch medical Research with Human Subjects Law (MEC-2018-1302), and all patients gave written consent. The study was funded by the Erasmus MC Sophia, Department of Neonatology and Obstetrics and Prenatal diagnosis and carried out according to The Code of Ethics of the World Medical Association (Declaration of Helsinki).

### Sample collection and storage

25 ml of midstream or catheter urine was collected within the study period from pregnant women at admission and stored at 4°C within 30 min in a 50 ml Falcon tube. Next, the samples were transported on ice, and restored at −20°C.

Shortly before sample preparation, the urine samples were defrosted in their original collection tube and manually mixed gently, no centrifugation or filtration was applied in order to prevent significant alterations in the urinary metabolome. Subsequently, 5 ml of each sample was transferred into glass vials (20 ml headspace vial, Thames Restek, Saunderton, UK) with a 3 ml transfer pipette (Thermo Scientific™, United States). The remaining urine samples were again frozen at −20°C for future analyses. The selected subsamples were immediately frozen again at −20°C. For analysis, they were shipped to the School of Engineering at the University of Warwick (Coventry, United Kingdom) on dry ice (−80°C). At arrival, the subsamples were immediately stored at −20°C before analysis, which took place in May 2019.

The urine samples were analyzed using gas chromatography coupled to an ion mobility spectrometer (GC-IMS, FlavourSpec®, G.A.S., Dortmund, Germany). This technique measures the VOCs emanating from the urine samples. The headspace, containing the urinary VOCs, was injected into the GC-IMS, where the VOCs were first pre-separated by means of GC according to methodology described in previous work (25). This separation is based on the interaction of the molecules with the column lining. The pre-separated VOCs then enter the GC-IMS where they are ionized by a low radiation tritium (H3) source. This creates reactant ions, which subsequently travel in an electric field against the flow of an inert drift gas at atmospheric pressure. The ionized molecules are selectively decelerated based on the interaction with the drift gas, before being detected. Thus, the molecules are separated depending on their size, charge and mass. The experimental conditions were as follows; the GC used a 15 m, SE-54 column (CS Chromatography, Germany) and was performed at 40˚C using nitrogen 99.9% (3.5 bar) as the carrier gas. The IMS used nitrogen as the drift gas and was performed at 45°C. The flow rate for the nitrogen was 20 ml/min (34.175 kPa) for 6 min (GC), and 150 ml/min (0.364 kPa) (IMS). From each urinary sample a unique chromatogram was created.

### Primary analysis

**Figure 1:**
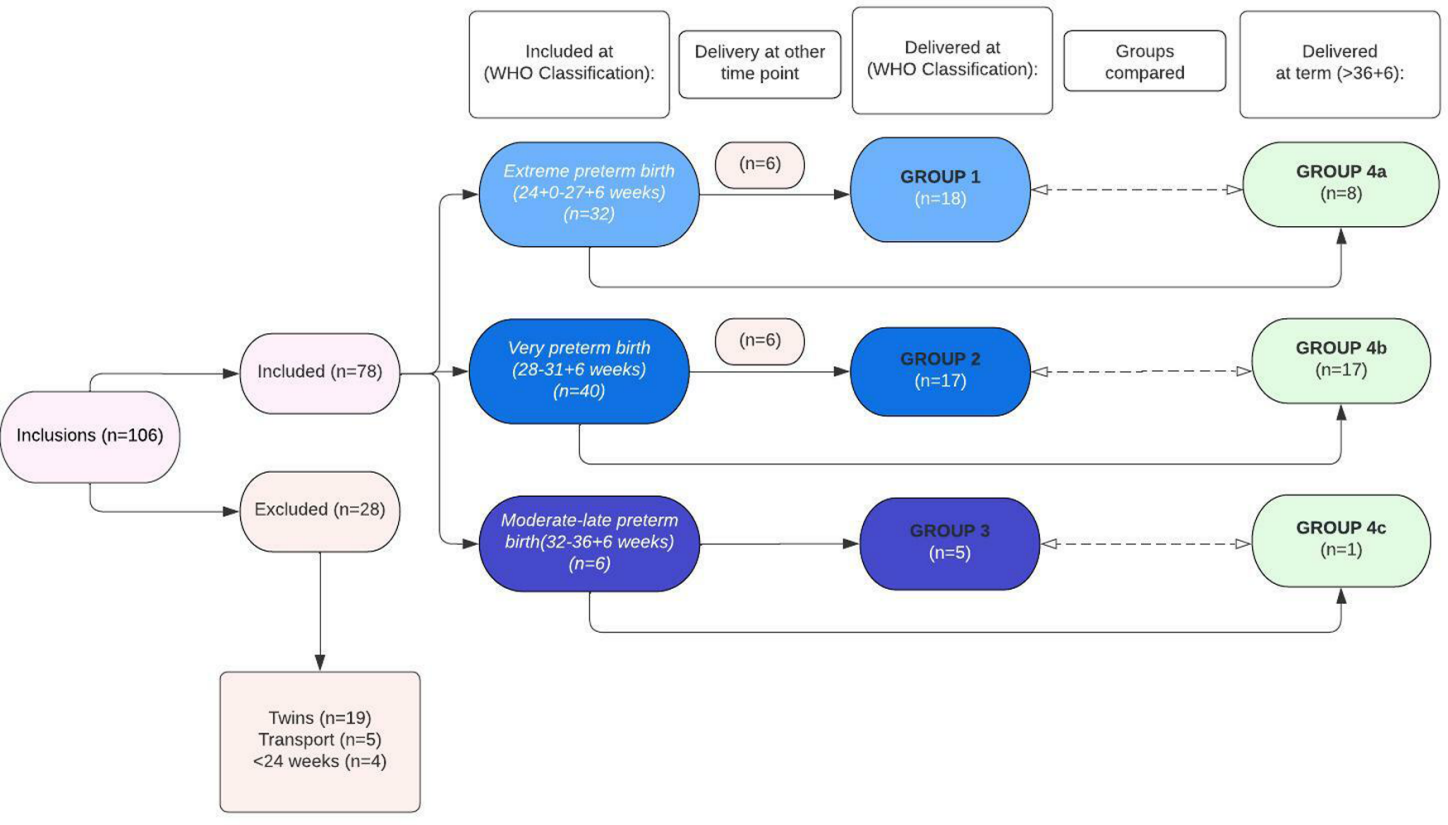
Flowchart showing inclusions and exclusions.

First, VOCs of urine samples collected at admittance of women who delivered preterm (<36 + 6 weeks) were compared with VOCs of urine samples of women admitted for suspicion of PB (24 + 0 to 36 + 6 weeks), but who ultimately delivered at term (>36 + 6 weeks of gestation), to determine specific differences in urinary VOC patterns between both groups. Secondly, sub-analyses were performed based on the WHO categories of preterm birth at inclusion and delivery, namely: (1) extremely preterm birth (included and delivered between 24 + 0 to 27 + 7 weeks; group 1), (2) very preterm birth (included and delivered between 28 + 0 to 31 + 6 weeks; group 2), and (3) moderate to late preterm birth (included and delivered between 32 + 0 to 36 + 6 weeks; group 3). These groups were all matched to women with an imminent preterm birth included at the same gestational age category, but whom eventually delivered at term (group 4a (included from 24 + 0 to 27 + 7 weeks), group 4b (included from 28 + 0 to 31 + 6 weeks), and group 4c (included from 32 + 0 to 36 + 6 weeks)) (Figure 1). Due to the low sample size in group 3 and group 4c (*n* = 6 and *n* = 1, respectively), these groups were combined with respectively group 2 and group 4b when performing the sub-analyses.

### Secondary analysis

Due to the multifactorial etiology of PB, additional parameters associated with preterm birth were included and analyzed. This analysis was performed comparing the urinary VOCs of women based on increased C-reactive protein (CRP) (cut-off > 9 mg/L as based on Erasmus MC criteria), cervical length (CL) (<25 mm as based on national guidelines (24), urinary tract infections (UTI; based upon a positive nitrite in the urine and/or a positive bacterial urine culture based on bacterial growth) and chorioamnionitis (based upon postpartum histopathological diagnosis of the placenta).

### Statistical analyses

Supplementary Figure S1 shows an example of a chromatogram from one of the urine samples, which is a typical output from a GC-IMS device. The colored peak areas show unique chemicals being detected by the GC-IMS instrument. Each data point is characterized by the retention time in the GC column and the drift time through the IMS. The intensity of the ion current signal, indicated by color, represents the level of VOC detected in the sample.

The GC-IMS output in Supplementary Figure S1 shows that each sample contains an extensive amount of chemical information, though with large areas of sparse data. Therefore, before statistical analysis, the data is pre-processed to reduce the dimensionality of the data set to around 10,000 non-zero data points. These datapoints comprise all the information and the chemical information in the sample. This is achieved by first “cropping” the central section of the output file, where all the chemical information is located. To this, a small threshold is applied, where all the values below the threshold are set to zero. The value of this threshold is selected to be just above the noise level of the instrument and the same crop and threshold values are used for all the sample files.

To reduce the risk of over-classification, class prediction was undertaken using a 10-fold cross-validation method. The data set is divided into 10 equal subsets, where 90% of the data is used as a training set and the remaining 10% as validation data for model testing. This was repeated 10 times until all the samples have been a test sample. Within each fold, a Wilcoxon rank sum test was used for calculating *p*-values in the training set to identify the important features. The 100 features with the smallest *p*-values were used to train different classifiers, specifically Sparse Logistic Regression, Support Vector Machine and Gaussian Process, which have been previously used in such clinical studies (26). These classifiers were applied to the test set, and this was repeated until every sample has been classified. From the resultant probabilities, statistical parameters, including receiver operator characteristic (ROC) curve [thus area under curve (AUC)], sensitivity, specificity, positive predictive value (PPV), negative predictive value (NPV) and *p*-values. The accuracy of the algorithms was determined by their AUC and *p*-value. The best performing classifier was chosen for every comparison. Demographic data was analyzed using SPSS. For continuous variables, ANOVA was used and for categorical variables, the Chi-squared test and Fisher's exact test were used (Table 1).

**Table 1:** Baseline characteristics of the whole study population (*n* = 78). Data are presented as median with standard deviations (SD) and significant results are presented in red.

	Total study population (*n* = 78)	Term (*n* = 26)	Preterm (*n* = 52)	*P*-value
Age in years	31.06 (5.21)	31.77 (5,13)	30.71 (5.27)	0.34
Ethnicity (*n* = 64)				0.16
Caucasian	39	10	29	
Non-caucasian	25	12	13	
Missing	14			
BMI in kg/m^2^	26.04 (5.55)	24.75 (5.80)	26.67 (5.34)	0.14
Length in cm	165.84 (6.77)	163.72 (6.45)	166.87 (6.74)	0.06
Weight in kg	71.79 (16.63)	69.52 (17.04)	74.65 (15.90)	0.05
Alcohol use, yes (*n*)	1	0	1	1.00
Smoking, yes (*n*)	6	2	4	1.00
Previous PB, yes (*n*)	21	5	16	0.34
Chorioamnionitis, yes (*n*)	28	0	28	<0.01
Positive vaginal swab, yes (*n*)	9	3	6	1.00
Positive urine culture, yes (*n*)	7	3	4	0.70
Maternal antibiotics use, yes (*n*)	18	7	11	0.62
PPROM, yes (*n*)	21	1	20	0.01
Cervical length in mm	20.81 (10.82)	21.46 (9.96)	20.54 (11.23)	0.65
CRP in mg/L	12.97 (14.43)	6.74 (7.09)	21.43 (17.59)	0.00
Prenatal corticosteroids, yes (*n*)	70	23	47	1.00
Mode of delivery (*n*)				<0.00
Spontaneus	44	13	31	
Induced	10	9	1	
C-section	24	4	20	
Tocolytics, yes (*n*)	63	21	42	0.76
Previous cervical surgery, yes (*n*)	3	0	3	1.00

## Results

### Patient characteristics

Table 1: Baseline characteristics of the whole study population (*n* = 78). Data are presented as median with standard deviations (SD) and significant results are presented in red.

### Primary analysis

No significant difference was found between the urinary VOCs of women delivering preterm overall (>24 + 0 weeks until <37 + 0 weeks; group 1–3) compared with the women who delivered at term (>37 weeks; group 4a-c) (*p*-value 0.36; AUC 0.56).

We found significant differences in urinary VOCs of women admitted between 28 + 0 and 31 + 6 weeks of GA who delivered very preterm (group 2, see Material and Methods) in comparison with the group of women delivering at term (group 4b) (*p*-value 0.01, AUC 0.74 (see Table 2). When group 2 (*n* = 17) was combined with women who were admitted between 32 + 0 until 36 + 6 weeks of GA (and who also delivered during this period, group 3 (*n* = 5), we also observed significant differences in urinary VOC profiles compared with those who delivered at term (groups 4b + 4c) (*p*-value 0.01; AUC 0.70).

**Table 2:** GC-IMS results for the discrimination of urinary VOC of pregnant women that delivered preterm vs. term.

Groups compared		Classifiers	*p*-value	AUC (CI 95%)	Sensitivity (±95% CI)	Specificity (±95% CI)	PPV	NPV
Preterm: 24 + 0 until 36 + 6	Term: >37 weeks	SLR	0.36	0.56 (0.45–0.68)	0.96 (0.92–1.00)	0.12 (0.03–0.23)	0.71	0.60
Group 1: 24 + 0 until 27 + 6	Group 4a: >37 weeks	SLR	0.99	0.78 (0.58–0.97)	0.63 (0.24–0.91)	0.83 (0.59–0.96)	0.63	0.83
Group 2: 28 + 0 until 31 + 6	Group 4b: >37 weeks	SLR	**0** **.** **01**	**0.74** (**0.59–0.87)**	**0.82** (**0.67–0.95)**	**0.94** (**0.82–1.00)**	**0**.**53**	**0**.**80**
Group 2 + 3: 28 + 0 until 36 + 6	Group 4b + c: >37 weeks	SVM	**0**.**01**	**0.70** (**0.54–0.85)**	**0.44** (**0.22–0.69)**	**0.93** (**0.76–0.99)**	**0**.**80**	**0**.**72**

VOC, volatile organic compounds; GC-IMS, gas chromatography – ion mobility spectrometry; AUC, area under the curve; CI 95%, confidence interval 95%; SLR, Sparse Logistic Regression; SVM, Support Vector Machine.

No significant difference was found between the extremely preterm (24 + 0–27 + 6 weeks; group 1) vs. the term group (>37 weeks; group 4a) (*p*-value 0.99; AUC 0.78).

When comparing preterm groups mutually, we found a significant difference between women delivering extremely preterm (group 1) vs. very preterm-late preterm (groups 2 + 3) (*p*-value 0.02; AUC 0.69) (see Supplementary Table S1). The corresponding ROC curves comparing the urinary VOCs of women who gave PB vs. term are shown in Supplementary Figure S2.

### Secondary analysis

The secondary analysis demonstrated that urinary VOC profiles allowed for discriminating pregnant women with chorioamnionitis from women without chorioamnionitis (*p*-value 0.001; AUC 0.72) and between women with and without a UTI (>0.001; AUC 0.97) (Table 3). A significant difference in VOC profiles was also detected based on cervical length at admission (*p* -value 0.027; AUC 0.68), while it was not possible based on CRP levels in the blood (Table 3). Supplementary Figure S3 shows the ROC curves for the secondary analysis.

**Table 3:** GC-IMS results for the post-hoc analysis.

Groups compared		Classifiers	*p*-value	AUC (CI 95%)	Sensitivity (±95% CI)	Specificity (±95% CI)	PPV	NPV
No chorioamnionitis (*n* = 50)	Chorioamnionitis (*n* = 28)	GP	**0** **.** **001**	0.72 (0.60–0.84)	0.80 (0.63–0.92)	0.60 (0.42–0.76)	0.67	0.75
No UTI (*n* = 71)	Confirmed UTI (*n* = 7)	GP	**>0**.**001**	0.97 (0.90–1.00)	1.00 (0.69–1.00)	0.90 (0.55–1.00)	0.91	1.00
CRP <10 mg/L	CRP >9 mg/L	GP	0.255	0.55 (0.41–0.68)	0.51 (0.34–0.69)	0.63 (0.45–0.79)	0.58	0.56
Cervix length >25 mm	Cervix length <25 mm	SLR	**0**.**027**	0.68 (0.51–0.86)	0.79 (0.54–0.79)	0.58 (0.33–0.80)	0.65	0.73

GC-IMS, gas chromatography – ion mobility spectrometry; AUC, area under the curve; CI 95%, confidence interval 95%; CRP, c-reactive protein; UTI, urinary tract infection; SLR, Sparse Logistic Regression; GP, Gaussian Process.

## Discussion

In this prospective, single-center, proof of principle cohort study, we observed that urinary VOCs did not allow for discrimination between women who delivered preterm (24 + 0 to 36 + 6 weeks of GA) from women who delivered at term. However, urinary VOCs did discriminate between women who delivered very preterm until late preterm (28 + 0 until 36 + 6 weeks of GA) from women who delivered at term, which includes the gestational period in which 95% of all preterm births occur. Importantly, based on the urinary VOC profiles, we were also able to discriminate between women with and without chorioamnionitis and urinary tract infections.

As a tertiary obstetrical and neonatal referral center, our patient population is mainly concentrated on women at risk for extremely and very PB (≧23 + 5 weeks until 32 + 0 weeks GA). Therefore, we decided to divide the total group of PB into the different WHO defined categories of PB. We noticed a significant difference in VOCs in urine samples matched for gestational age at collection in women delivering between 28 + 0–36 + 6 weeks of gestation vs. delivery at term. Although tertiary care is aimed at <32 + 0 weeks of gestation, adequate detection of PB in the very preterm till late preterm category is of utmost importance to provide proper perinatal and prevent unnecessary fetal exposure to antenatal corticosteroids. Also, late PB is important to diagnose since approximately 85% of PBs occur between 32 + 0–37 + 0 weeks of gestation, with 10% occurring between 28 and 32 weeks (2).

The significantly different VOC profiles of women who delivered extremely preterm compared to the women who delivered very preterm until late preterm, suggests a difference in the (patho)physiologic and metabolic state of these individuals and could imply different etiologies between extremely PB and the remaining PBs. Literature from the developing countries shows that factors such as maternal undernourishment and anemia are independently associated with extreme PB (27). Unfortunately, literature from developed countries is lacking. Cervical insufficiency due to lack of supportive tissue like collagen, occurring when the cervix begins to efface and dilate during the second trimester, happens to about 1 percent of women and does not have an infectious cause. Both gynecological history, such as the removal of precancerous lesions, and previous pregnancy losses increase the risk for cervical insufficiency in a future pregnancy and are often treated with a cervical cerclage to help prevent premature birth (28).

However, another recent study into prediction of PB (26), showed that VOCs from vaginal swabs obtained closest to delivery analyzed by GC-IMS were most predictive for PB with a positive predictive value of up to 86%. These results indicate that VOCs from vaginal swabs change longitudinally during pregnancy, which could also be in line with our findings of differences in VOC profiles between the different gestational categories.

Microbiological studies into sPB show that intrauterine infections, such as chorioamnionitis, account for about 25%–40% of sPBs, while disturbances of the vaginal microbiome (leading to bacterial vaginosis) and urinary tract infection (UTI), affecting 8% of all pregnant women, are known risk factors for PB (2, 6, 29, 30). In our study, 36% of the women could be identified with chorioamnionitis and 9% with UTI based on VOCs (respectively, AUC 0.72, sensitivity 0.80 and specificity 0.60), which is in line with the literature (30). Until now, the diagnosis of UTI based upon positive bacterial urine culture takes several days, while chorioamnionitis can only be determined postnatally. A previous study, based on VOCs of exhaled breath, demonstrated that pregnant sheep intra-amniotically inoculated with *Ureaplasma parvum* could be classified as having chorioamnionitis compared to those without (with a sensitivity of 0.83, a specificity of 0.71 and an AUC of 0.93) (22). Also, UTI samples could be distinguished from non-UTI samples based on VOCs (16). These and our results indicate that non-invasive detection of the presence of intra-uterine or urinary tract infection is possible based on the use of different VOC profiles. Bedside antenatal detection of UTI or chorioamnionitis could lead to quickly applicable antibiotic treatment or a personalized decision to induce labor, lowering the risk of prolonged intra-uterine infection and detrimental effects for both mother and fetus.

Strengths of our study include the prospective study design, the standardized method of sample collection and short processing time preventing possible contamination or changes in VOCs. Another strength is that our group has previous experience in analysis of urinary VOCs in detecting different disease states (31, 32). Furthermore, baseline characteristics between the preterm and term groups were similar, except for the presence of chorioamnionitis, PPROM, elevated CRP and mode of delivery (favoring C-section) in the preterm groups. Limitations include relatively small sample sizes per WHO category (despite >100 initial inclusions), which prevented subgroup analysis (e.g., for gestational diabetes or pre-eclampsia), and necessitates validation in a larger cohort. The positive predictive value and negative predictive values of our test for groups 2 and 3 were relatively low (ranging from 0.53 to 0.80), also highlighting the importance of external validation. The inclusion of women with a UTI or chorioamnionitis, may influence the baseline VOCs, leaving more subtle VOC differences unnoticed. Another potential limitation is the freeze-thaw cycle, possibly influencing optimal sampling such as described in fecal VOC sample preparation (21, 25).

In a future study, aliquoting urine samples should be considered, as should standardization of sampling time (such as first-morning urine), and taking confounders such as diet, diuresis and exercise into account. Further optimization of VOC analysis in urine could be achieved by using auto-samplers (21). However, the potential clinical applicability would substantially suffer from a once-daily collection since the occurrence of preterm birth can happen anytime, and would prevent spot-on diagnostics in the future, making the method more time-consuming and less valuable in clinical practice. Furthermore, most errors in urinalysis have been described in the pre-analytical stage, highlighting the need for a well-established and universal method regarding collection, transportation and sample preparation (21, 33).

Overall, in the investigated group, 31% of pregnant women were unnecessarily admitted to the hospital and exposed to corticosteroids and tocolytics. Albeit in The Netherlands, antenatal corticosteroids to improve fetal lung maturation are indicated until 33 + 6 weeks of GA, a suspicion of preterm birth after 33 + 6 weeks of GA still results in unnecessary hospital admissions. Adequate and timely prediction of PB will result in targeted administration of antibiotics and antenatal corticosteroids, reduced duration of admission, timing and mode of delivery, and a decrease in admission to an inpatient ward. Importantly, this will lead to a reduction in health care costs and side effects of medical treatment. Administration of antenatal corticosteroids increases maternal leukocyte and fasting glucose levels, and after multiple courses, pregnant women are at risk for infections. Neonatal side effects include a reduction of fetal heart rate variation, multiple courses possibly affecting intrauterine growth and birth weight (34). Moreover, unnecessary fetal exposure to corticosteroids could have effects along the postnatal life course. A recent retrospective cohort study on the effects of antenatal corticosteroid treatment during postnatal development showed a significantly higher cumulative incidence and hazard rate for mental and behavioral disorders in antenatally unnecessarily exposed children (8, 9).

VOC analysis by GC-IMS is characterized by pattern recognition rather than identification of individual compounds. Our results highlight the need to identify the specific metabolites responsible for differences in urinary VOCs characterizing PB, which are probably related to factors such as inflammation, immunology, microbiota, diet or a combination of these factors (35). Although it is known that the vaginal microbiome of women changes during pregnancy (36), it has not yet been possible to use the vaginal bacterial compositions at any point in pregnancy to predict PB. The effect and influence of the dynamics of steroid hormones on VOC composition could also be of interest since a recent study illustrated that maternal 11-deoxycorticosterone serum levels were significantly associated with spontaneous PB <32 weeks (AUC = 0.77) (37). Identification of discriminative VOCs on a molecular level should be the focus of future studies which could then be used to investigate the etiology and mechanism of spontaneous PB and for prediction of PB. Implementation of a combination between non-invasive, bedside analysis of urinary and vaginal VOCs, in combination with for example fetal fibronectin and cervical length measurements, could prove extremely beneficial.

In conclusion, this proof of principle study shows that analysis of urinary VOCs seems to discriminate between women who deliver very preterm until late preterm from women who are suspected of preterm birth but will deliver at term. Furthermore, UTI and chorioamnionitis may be quickly, non-invasively and antenatally detected based on urinary VOCs. Early, accurate prediction of PB may allow for targeted intervention, improving perinatal outcome. Our results need to be externally validated in a larger cohort before implementation in clinical practice.

## Data Availability

The original contributions presented in the study are included in the article/**Supplementary Material**, further inquiries can be directed to the corresponding author.
