# Supplementary material for: Detection of spontaneous preterm birth by maternal urinary volatile organic compound analysis: A prospective cohort study
Source: Front Pediatr. 2022 Dec 12;10:1063248. doi: 10.3389/fped.2022.1063248 (PMC9791099; doi:10.3389/fped.2022.1063248)
Supplement: Supplementary file 1 [file Datasheet1.pdf]

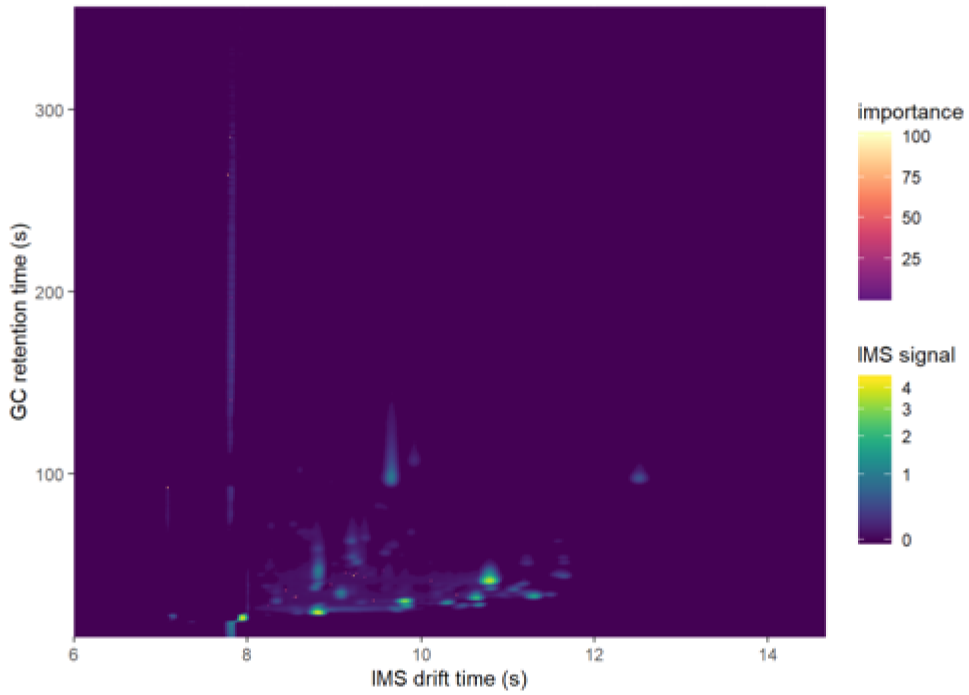

Supplemental Figure S1. Typical output (chromatogram) from the GC-IMS instrument from one of the urine samples

Supplemental Table S1. **GC-IMS results for the discrimination of urinary VOC of women who gave preterm birth at different gestational ages.**								
Groups compared		Classifiers	p-value	AUC (CI 95%)	Sensitivity (± 95% CI)	Specificity (± 95% CI)	PPV	NPV
Group 1: 24+0 until 27+6	Group 2: 28+0 until 31+6	SLR	0.05	0.67 (0.53-0.81)	0.62 (0.44-0.79)	0.59 (0.42-0.76)	0.62	0.59
Group 1: 24+0 until 27+6	Group 2+3: 28+0 until 36+6	SVM	0.02	0.69 (0.53-0.85)	0.56 (0.31-0.78)	0.79 (0.59-0.92)	0.63	0.73
Abbreviations: VOC, volatile organic compounds; GC-IMS, gas chromatography – ion mobility spectrometry; AUC, area under the curve; CI 95%, confidence interval 95%; SLR, Sparse Logistic Regression; SVM, Support Vector Machine								

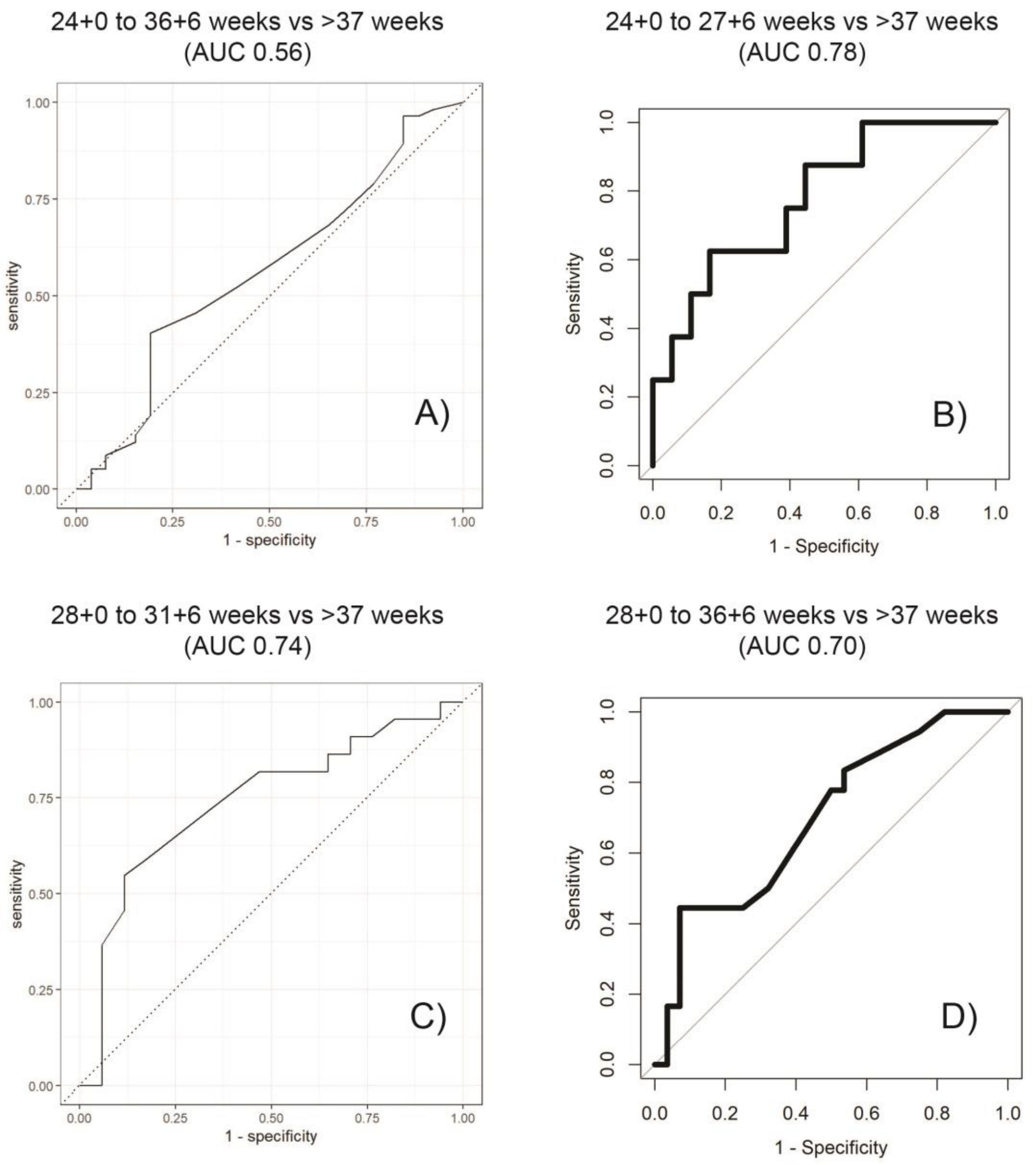

Supplemental Figure S2: Receiver operator characteristic using GC-IMS for (a) all preterm vs term; (b) 24+0 to 27+6 weeks vs >37 weeks; (c) 24+0 to 31+6 weeks vs >37 weeks; and (d) 28+0 to 36+6 weeks vs >37 weeks.

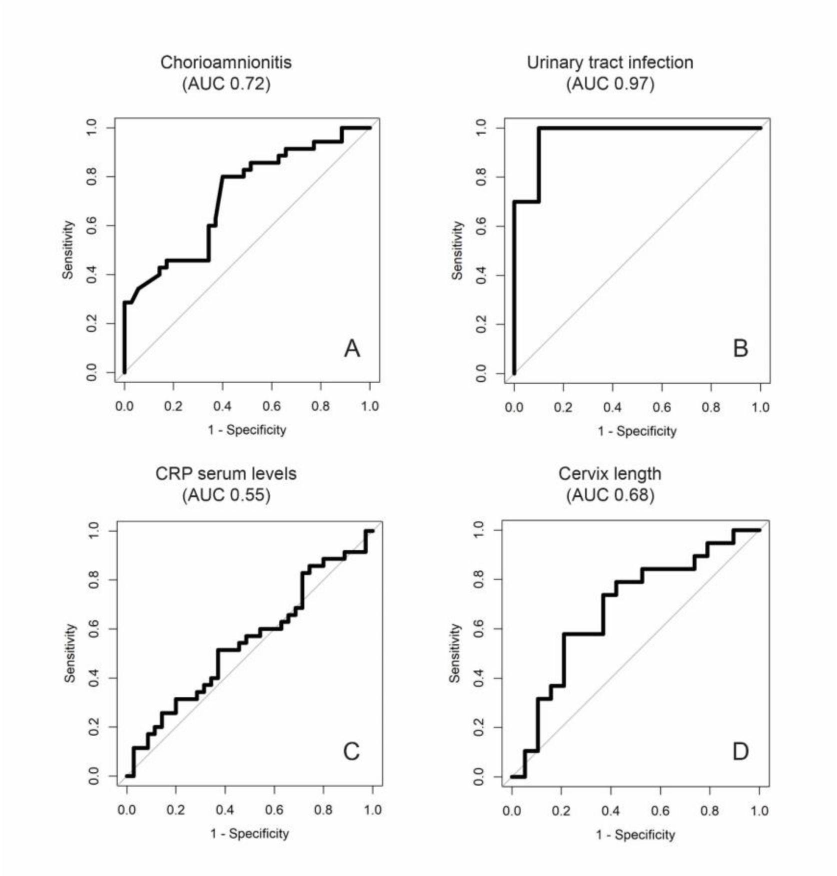

Supplemental Figure S3: Receiver operator characteristic using GC-IMS for (a) chorioamnionitis vs no chorioamnionitis; (b) confirmed urinary tract infection vs no urinary tract infection; (c) CRP levels >9 mg/L vs CRP <10 mg/L; and (d) a cervix length of >25mm vs a cervix length of <25mm.
